# “Damaged genitals”—Cut women's perceptions of the effect of female genital cutting on sexual function. A qualitative study from Sweden

**DOI:** 10.3389/fsoc.2022.943949

**Published:** 2022-08-12

**Authors:** Malin Jordal, Jessica Påfs, Anna Wahlberg, R. Elise B. Johansen

**Affiliations:** ^1^Department of Caring Sciences, University of Gävle, Gävle, Sweden; ^2^Department of Social Work, University of Gothenburg, Gothenburg, Sweden; ^3^Department of Women's and Children's Health, Akademiska Sjukhuset, Uppsala University, Uppsala, Sweden; ^4^Norwegian Centre for Violence and Traumatic Stress Studies, Department of Children and Adolescents, Oslo, Norway

**Keywords:** clitorectomy, clitoral reconstruction, defibulation, excision, female genital cutting, infibulation, sexual function

## Abstract

Female genital cutting (FGC) is a traditional practice, commonly underpinned by cultural values regarding female sexuality, that involves the cutting of women's external genitalia, often entailing the removal of clitoral tissue and/or closing the vaginal orifice. As control of female sexual libido is a common rationale for FGC, international concern has been raised regarding its potential negative effect on female sexuality. Most studies attempting to measure the impact of FGC on women's sexual function are quantitative and employ predefined questionnaires such as the Female Sexual Function Index (FSFI). However, these have not been validated for cut women, or for all FGC-practicing countries or communities; nor do they capture cut women's perceptions and experiences of their sexuality. We propose that the subjective nature of sexuality calls for a qualitative approach in which cut women's own voices and reflections are investigated. In this paper, we seek to unravel how FGC-affected women themselves reflect upon and perceive the possible connection between FGC and their sexual function and intimate relationships. The study has a qualitative design and is based on 44 individual interviews with 25 women seeking clitoral reconstruction in Sweden. Its findings demonstrate that the women largely perceived the physical aspects of FGC, including the removal of clitoral tissue, to affect women's (including their own) sexual function negatively. They also recognized the psychological aspects of FGC as further challenging their sex lives and intimate relationships. The women desired acknowledgment of the physical consequences of FGC and of their sexual difficulties as “real” and not merely “psychological blocks”.

## Background

Female genital cutting (FGC) is the physical alteration of women's external genitalia, often involving cutting the clitoris and/or labia, or narrowing the vaginal orifice (WHO, 2008). The World Health Organization (WHO) typically divides FGC into four types: Type I involves partial or total removal of the clitoris and/or the prepuce (clitorectomy); Type II entails partial or total removal of the clitoris and the labia minora, with or without excision of the labia majora (excision); Type III involves a narrowing of the vaginal orifice with the creation of a covering seal, with or without excision of the external parts of the clitoris (infibulation); and Type IV refers to all other harmful procedures performed on the female genitalia for non-medical purposes, such as pricking, piercing, incising, and scraping (WHO, 2008). Around 200 million women and girls worldwide have undergone some form of cutting (UNICEF, 2016). The practice is most prevalent in countries and regions in Africa, the Middle East, and Asia, but has become a global phenomenon due to migration (WHO, 2008). Despite years of anti-FGC campaigns aimed at eradicating the practice, the prevalence of FGC has declined only marginally; in fact, in actual numbers it is believed to be increasing due to population growth (UNICEF, 2016). An estimated half a million women and girls with FGC live in Europe (Van Baelen et al., 2016), 38,000 of them in Sweden (The National Board of Health and Welfare, 2015).

The cultural meaning of FGC varies between communities and over time, but a common cultural underpinning is control of women's sexual libido (Berg and Denison, 2013). While infibulation signifies an external “hymen” ensuring virginity prior to marriage, some studies have found the rationale for clitorectomy to be based on a perception of the clitoris as the site of women's sexual drive, which thus has to be cut to ensure their sexual morality (Johansen, 2016). This rationale has raised concern regarding the potential negative effects of FGC, particularly clitorectomy, on female sexuality. While negative health consequences after FGC—including obstetric, psychological and sexual problems—are widely reported (Berg et al., 2010, 2014; Berg and Denison, 2012; Villani, 2022), studies investigating the effects of FGC on sexual function have inconsistent or contradictory findings. This is largely due to difficulties involved in measuring sexuality in finding an appropriate comparison group as well as the complex interplay between physical, psychological and sociocultural aspects of sexuality (Esho, 2012; Johnson-Agbakwu and Warren, 2017). Thus, some studies find increased risk of impaired sexual function among women who have undergone FGC (Esho et al., 2017; Rouzi et al., 2017; Buggio et al., 2019; Pérez-López et al., 2020; Nzinga et al., 2021) while others do not (Obermeyer, 2005; Catania et al., 2007; Abdulcadir, 2016). Many of these studies, however, do not distinguish between the different types of FGC or variations in the anatomical extent of the cutting.

Impaired sexual function is characterized by difficulty moving through the stages of sexual desire, arousal, and orgasm, but also involves the subjective experience of sexual satisfaction (Rosen et al., 2000). Many of the existing studies investigating the effects of FGC on sexual function have used predefined questionnaires such as the Female Sexual Function Index (FSFI) (Catania et al., 2007; Ismail et al., 2017; Rouzi et al., 2017; Pérez-López et al., 2020; Nzinga et al., 2021). The FSFI is a well-used tool for measuring desire, subjective arousal, lubrication, orgasm, and pain (Rosen et al., 2000), but is not adapted to or validated for use among women with FGC or for many of the various cultural settings women with FGC belong to. Further, the instrument has been critiqued for failing to explore the socio-cultural factors involved in women's experiences of sexual function. Johnsdotter (2020, p. 13) writes about FSFI that it is “is a blunt instrument for capturing sexual experiences—and it completely overlooks social and cultural factors that affect how we experience such elusive bodily sensations as sexual desire, satisfaction and pain”. Thus, the FSFI is likely to be insufficient in investigating women's subjective perceptions and experiences of a potential connection between FGC and sexual function.

Villani (2022) notes that questions of pleasure and desire are largely embedded in social expectations and norms, which should be considered when studying the sexual consequences of FGC. It has been argued that cut women's encounter with Western values—which tend to assign higher significance to women's sexual rights to desire and pleasure, and to the importance of the clitoris in securing these things –affects their perceptions of their own sexuality and its relation to FGC (Johnsdotter, 2013; Ziyada et al., 2020; O'Neill et al., 2021). A more thorough understanding of the complexity behind cut women's understanding and meaning-making of the potential connection between FGC and sexual experiences, including the socio-cultural-symbolic nexus (Esho, 2012), could inform care providers, sex counselors, policy-makers, and others aiming to provide healthcare for this group of women. To contribute to this research gap, we aim to explore whether and how cut women residing in Sweden perceive that FGC has affected their sexual function and intimate relationships.

## Methods

### Design, recruitment, and data collection

The study has a qualitative design, which is useful when endeavoring to explore a complex and underresearched phenomenon (Kvale and Brinkmann, 2009) such as cut women's perceptions and experiences of sexual matters. The inclusion criterion was having undergone FGC. All the women were recruited at the Karolinska University Hospital in Sweden upon requesting clitoral reconstructive surgery, which is aimed at improving the anatomy and function of the clitoris (Foldès et al., 2012). The findings of this article thus derive from a larger data set exploring motives and expectations for, and experiences of, clitoral reconstructive surgery. The women were asked by the surgeon or a psychosexual counselor to consent to being contacted for the study. If interested, they were contacted by the first author and given an information letter stating the study's aim and purpose. Of the 27 women who replied to the first author's contact, 25 agreed to participate in the study. Twenty-two of these women ultimately underwent clitoral reconstruction, while three of them decided not to go through with it.

Semi-structured interviews were used to collect the data. The interviews were conducted during the period 2016–2019, and lasted 23–80 min. They were carried out in the participant's home, in a private room on the hospital premises, or at a library or a cafe, depending on practicalities and the woman's preferences. Eighteen of the participants were interviewed twice: first upon requesting surgery and then about 1 year post-surgery. The three women who declined surgery were interviewed for the second time after having made this decision. In total, 44 interviews were conducted.

The interviews started with the interviewer obtaining informed consent and informing the woman about measures for ensuring confidentiality, that participation was voluntary, and of her right to withdraw from the study at any point without explanation as well as to decline to answer any questions if she felt uncomfortable. The first interviews (upon requesting clitoral reconstructive surgery) focused mainly on the motives for requesting and expectations for the surgery. However, these interviews also explored the women's memories and perceptions of their FGC, and their genital, mental, and sexual concerns, particularly related to pain, sexual function, body (genital) image, identity, and relational factors. The second interview focused mainly on the after-effects of the surgery, particularly related to pain, sexual function, body (genital) image, identity, and relational factors; or, if they declined surgery, their reasons for changing their mind. The findings around these questions are reported elsewhere (see author and author); thus, the present paper relies solely on data related to the women's perceptions and experiences of the potential effects of FGC on sexual function, including their own. Three of the interviews were conducted in English, two in Somali using an interpreter (physically present or by telephone), and the remaining 39 in Swedish. Thirty-eight interviews were conducted face-to-face and six over the telephone. Forty interviews were audio-recorded and later transcribed, while in the remaining four the interviewer took notes due to technical problems or the woman not feeling comfortable being recorded. Here, more detailed transcripts were written down immediately after the interviews with help of the notes. The study was approved by the Regional Ethical Review Board in Stockholm (2015/1188-31). Descriptions of personal characteristics are kept to a minimum, and pseudonyms are used for all participants to protect the women's confidentiality.

### Reflection of the position as an interviewer

The qualitative research interview is a co-creation between interviewer and interviewee (Peeck, 2016). Asking cut women about FGC and sexual difficulties may be uncomfortable for both parties, but particularly for the interviewee. A first step for minimizing such discomfort was to emphasize the voluntariness of the study; if a woman did not respond to the first contact attempted by the researcher, this was interpreted as a wish to refrain from participating. If the woman agreed to be interviewed, however, the interviewer paid significant attention to establishing rapport and to making the interview situation as comfortable as possible. This involved emphasizing the conversational character of the interview. Also, the interviewer sought to maintain an empathetic, non-judgmental attitude, which involved being sensitive to signs of discomfort when discussing sexual matters. While encouraging the women to freely express their opinions, feelings, and experiences, efforts were made not to push them to talk. Consequently, the interviewed women's accounts of sexual matters varied; while all of them were asked how they viewed the connection between FGC and sexual function, some avoided the topic or spoke about it in very general terms, while others talked more openly and included personal experiences. Further, the researcher's position as a white, uncut woman may have intimidated some of the women as they positioned the researcher as belonging to a group of women with “intact” genitals and thus distinguished from themselves. This was sometimes indicated, for example by a woman referring to the interviewer as being among “those of you who have clitorises”. To balance out a potential sense of difference between interviewer and interviewee (Liamputtong, 2010), the interviewer endeavored to avoid supporting the narratives of “FGC damages sexual function” and “uncut women have problem-free sex lives”. Instead, the interviewer attempted to interrogate these matters openly and non-judgmentally, sometimes also clarifying that sexual problems existed among uncut women as well. While some women visibly found it difficult to talk about certain sexual matters, which they expressed through bodily manifestations such as embarrassed laughter, looking down, or refraining from answering certain questions, others conveyed a sense of relief at being able to discuss such matters with a non-judgmental listener.

### Data analysis

Thematic analysis was used to analyze the data (Braun and Clarke, 2006). First, all interview transcripts were read thoroughly several times, looking for content related to the study objectives, which was highlighted and extracted. Subsequently, these excerpts were coded and organized into themes summarizing the essence of the data. The themes were worked and reworked until they provided a sound and clear demonstration of the interview content reflecting the study objectives. Thus, the process was a dynamic and non-linear movement involving reading and re-reading the whole data set, excerpts, codes, and themes, until a sense of having captured the core meaning of the data had been reached (Braun and Clarke, 2006).

### Characteristics of participants

All the participants were first-generation immigrants in Sweden. They had migrated from Eritrea (*n* = 4), the Gambia (*n* = 2), Iraq (*n* = 2), Senegal (*n* = 1), Sierra Leone (*n* = 2), and Somalia (*n* = 14) either along with their families, through family reunification, or as sole migrants, many in childhood or early adulthood. Nineteen of them had lived in Sweden for 10 years or longer. The participants were aged 19-56 years at the first interview, with the majority in their 20s (*n* = 6) or 30s (*n* = 14). Many (*n* = 15) of them worked in the healthcare sector, primarily as nurses or nurse assistants. Others worked as engineers, personal assistants, or cleaners, or were studying. Seven of the women were married, three were divorced, and 15 were unmarried. Several of the divorced and unmarried women had a boyfriend. All women identified as heterosexual, and only three of them said they had never had sex. Eleven of them had children, and one had grandchildren.

Sixteen women reported having undergone FGC Type III, seven Type II, and two Type I. The majority with Type III (infibulation) had been defibulated prior to seeking clitoral reconstructive surgery, when they had given birth or on other occasions. The women had been cut at different ages: from infancy up to 9–10 years of age. While those who had been cut at a very young age could not remember the incident, others remembered their cutting as traumatic. Some had been given anesthesia and did not remember the actual cutting as traumatic but more so the healing process, which they recalled as having been painful.

## Findings

Almost all the interviewed women perceived that FGC has a negative effect on sexual function. They discussed learning that the purpose of FGC is to reduce women's sexual desire and enjoyment, and reading literature on the importance of the clitoris for women's sexual pleasure and orgasm. This made sense to them, as they themselves experienced FGC as having negatively affected their sexual experiences. While the women mainly believed that their impaired ability to enjoy sex had been caused by the physical alteration to their genitalia, particularly infibulation and the removal of genital tissue, they also regarded the psychological aspects of FGC to have caused difficulties in their sex lives and intimate relationships.

### Coming to understand the connection between FGC and sexuality

Many of the interviewed women said that in their adolescence or early adulthood they had come to understand that FGC was carried out to control women's sexuality. Some said that they had come to this understanding in the context of origin, others after coming to Sweden. Behar, a 46-year-old woman from Iraq, said she had come to realize this when growing up: *When I grew up, I understood that it [FGC] was to reduce women's sexuality*. The women reasoned that the main purpose of FGC was to diminish the woman's sexual libido in order to make her less promiscuous. Lola, a 32-year-old woman from Eritrea, said: *I guess it [FGC] is a way to hinder the woman from feeling pleasure when she's with a man; it must be due to that. Or that she should stay with one man, I don't know. But in some way, one wants to deprive the woman of her capacity to feel sexual pleasure, for her not to become sexually excessive*.

Some of the interviewed women had read books by female authors known for writing about their own experiences of FGC, such as Nawal el Saadawi and Waris Dirie. While the women largely perceived such literature as educative, they also recounted that it made them anticipate problems in their own sex lives.

As the women had migrated to Sweden or other Western contexts with liberal sexual rights, often in their childhood or early adulthood, many recounted how this exposure to Western culture had formed their understanding of FGC as “wrong” and “harmful” and as negatively affecting their capacity to enjoy sex. Amina, a 46-year-old woman from Somalia, said: *Because when I was young, you know, I knew nothing about sex, but then once you grow up and you read about sex in Cosmopolitan magazines (laughs a little) and you realize there's more to it than what you, than what you feel or experience…*

Being around female friends who openly discussed sexual pleasure and women's rights made the women reflect on their own sexuality. Sara, a 32-year-old woman from Somalia who had come to Sweden as a child, said: *We grew up in the 90s and there were so many girls' bands, and then it was a lot about owning your sexuality, eh, to feel pleasure during sex, and it was important not to be doing something you didn't enjoy…* In contrast to Sweden, where women were expected to enjoy sex for their own sake, many women described how sex in their own cultural background or upbringing was endorsed only within marriage and as a means to produce children. The interviewed women had come to distance themselves from such an ideal, which they considered old-fashioned and misogynistic. Instead, they had come to value women's right to sexual pleasure as an essential human right, which they saw as natural once they had been exposed to feminist and liberal thinking. Sara continued: *I also think this is natural, something that naturally comes when one enters, when one is exposed to more liberal thoughts… I think there's no woman who stands for women's rights who doesn't think she should also have that right [to sexual pleasure]. For me it's more of a feminist idea existing all over the world, even if the pressure comes, yes, it becomes more real because I live here [in Sweden]*.

Living in Sweden, many of the interviewed women compared themselves to non-cut women, who they considered to have a “normal” or “intact” sexuality. While not necessarily believing that uncut women could not experience sexual problems, they talked about these women's sexual function as contrasting with their own. Uncut women were perceived as having a natural ability to feel sexual desire and pleasure and to reach orgasm. Ruquia, a 37-year-old woman from Somalia, said:

*Ruquia: Well, that women have this need [for sex], I think this need, it's like when you're hungry, you need food. And when you have sexual desire, then you need someone. That's what I think*.

*Interviewer: But this isn't something you feel that you have?*

*Ruquia: No. But I don't think it's strange that others have it. I think it's normal*.

### Complex causes of FGC on the body, mind, and sexuality

When asked what they thought lay behind the potential connection between FGC and women's impaired sexual function, the women mainly related it to the physical alteration of the genitals caused by the cutting. Infibulation was said to make penetrative sex directly painful, and most of the interviewed women who had initially undergone infibulation described sex when infibulated as “horrible”, especially in the beginning. Ruquia said: *In the beginning it [sex] hurt a lot. After a while it became what it was, but it's nothing I enjoy or long for*. Even if sexual intercourse had become more manageable with time or after (partial) defibulation, many women described experiencing continued pain and discomfort during sexual intercourse. Aisha, a 56-year-old woman from Somalia, talked about difficulty having penetrative sex due to scars and an inelasticity of her genital tissue, even though she had been defibulated: *It's easy to get tears. If you have penetrative sex, you might have to stay away the whole week afterward so that it can heal*. Some said that even if they could initially experience sexual desire and excitement, this would turn into anxiety when they got closer to the actual sex as they anticipated that it would be painful.

While infibulation was said to make penetrative sex painful, the women also believed that the removal of clitoral tissue reduced women's capacity to feel sexual pleasure. Lola, a 32-year-old woman from Eritrea, referred to the scientific body of literature highlighting the importance of the clitoris for achieving orgasm when reasoning around cut women's sexual dysfunction: …*There are studies saying that most women have an orgasm after stimulating the clitoris. (…) If most women experience sexual enjoyment and have their orgasm through stimulus of the clitoris, how is it for the woman who doesn't have a clitoris; what should she be stimulated from? Or get this sexual pleasure?*

Some said they had realized the importance of the clitoris when they became sexually active. Fatou, a 30-year-old woman from the Gambia, discussed coming to this realization when she began having sex and experienced little pleasure: *Before I didn't know, because I don't know how, how it's so important for you to have your clitoris, I didn't know it before, because I was like, you know in my country they don't talk about sex in public (…) So I didn't know the importance of clitoris, until I started having sex…*

Several women reported limited genital or clitoral sensation and associated this with being cut. Ami, a 37-year-old woman from the Gambia, recounted the sensation of touching her genitals: *It feels like touching my elbows*. Others said they had “some sensation” in the clitoral area and had learned to achieve orgasm through masturbation by “pressing a little bit harder”. While most women complained about reduced clitoral sensation, two women recounted the opposite problem, describing an oversensitivity in the clitoral area. This was said to cause pain and discomfort when walking, touching oneself, or during sex. Zara, a 31-year-old woman from Iraq, said: *I had read that [cut] women are deprived of all the sensation, but I had sensation, even so much that it hurt when I touched myself*.

Thus, the women both assumed and experienced pain and an absence of genital sensitivity, which they connected to the physical alteration caused by FGC, which most of them regarded as the main cause of cut women's sexual problems. Yet, they also acknowledged psychological aspects related to having “damaged” genitals, which created shame and negatively affected their self-confidence. The combination of feeling unable to enjoy sex, having an inability to relax, and experiencing negative anticipation was demonstrated in the conversation with Ami:

*Interviewer: How do you think that what happened to you, the FGC, affects your sexuality?*

*Ami: It affects everything*.

*Interviewer: You think so?*

*Ami: It does; I don't “think”—it does*.

*Interviewer: Because you feel that it does? (Ami: mmhmm [signifying agreement]) You feel it physically? (Ami: Mmhmm) Do you think everybody who's gone through it (FGC), that no one experiences sexual enjoyment, that it doesn't work, or…?*

*Ami: For me it doesn't work*.

*Interviewer: It doesn't? Can you, I don't know if it's possible, but can you say something about what happens, why it doesn't work? Is it something physical, or is it something…?*

*Ami: I'm afraid, so like I'm afraid, they can't be down there, I can't relax, and then I don't believe that… It doesn't work*.

For Ami, the physical became psychological, and these two aspects in combination negatively affected her ability to enjoy sex. While few women fully dismissed the physical aspect of FGC as causing women's sexual problems, Leila, a 32-year-old woman from Somalia, was one who did. She had requested surgery mainly to restore her genitals aesthetically, not because she felt unable to enjoy sex, and rejected the assumption that FGC removed women's capacity to enjoy sex. Instead, she replied *It's all in your head* when asked about what she thought about cut women's complaints over sexual difficulties.

Others disagreed with such statements, and voiced frustration at what they considered a tendency to reduce cut women's sexual problems to “psychological blocks”. Lola said: *It's easy to say it's a psychological block, like “you don't have your clitoris and now you're psychologically blocked by that”. Of course, but it's still related to the physical; I really want to emphasize that. It's related to the physical: You don't feel it, you get no stimulus there. (…) And you think about it and the physical becomes psychological. And that, of course, becomes a block*.

While all the women had initially requested clitoral reconstruction surgery, some had come to reconsider their initial assumption that cut women's sexual problems were merely related to physical aspects. Sara had changed her mind after taking part in the sexual counseling offered in connection with the clitoral reconstruction, and had come to question her previous assumption that her sexual problems were related to the physical aspects of FGC. She said: *Starting to talk about it, accepting yourself, can make you feel less shame. Having difficulty having an orgasm may not only have to do with that [the physical consequences of FGC]*.

### FGC and its negative affect on intimate relationships

The interviewed women believed that difficulties experiencing sexual pleasure caused struggles in their intimate relationships. While some said they had largely stayed away from men, mainly due to shame or a fear of engaging in sexual relations, others said they endured sex for the sake of their partner. The women perceived their inability to enjoy sex as creating feelings of sadness, shame, and distrust. In turn, they felt these feelings negatively affected their ability to relax during sex with their intimate partner. Some said that their lack of interest in sex might push their partner to be unfaithful, which created a fear of abandonment and rejection. Amina, a 46-year-old married woman from Somalia, said:

*Interviewer: How would you… evaluate that relationship with him [your husband]?*

*Amina: Ehm… I think it's, I think we could look at it two ways, because we have children, we, the relationship is strong because of that. But I think if it were only based on sexuality [laughs a little], I think he would've left me a long time ago because he's, I feel he hasn't, I've denied him. Because I.. Yeah. I don't, ehm, it's, I'm not always easily.. sexually… [silence]*

*Interviewer: Yeah, I understand. Has he complained about that?*

*Amina: He has complained, he has and, you know, it's also interfered a little with our relationship because he's then had to look elsewhere. It hasn't been easy…*

Even if the women did engage in sex with their partner, they believed that the partner was able to tell that they did not enjoy the sex, which again made them feel guilty as they believed that this made the men enjoy it less. Ami said*: I feel ashamed. And then I feel bad, I feel sorry for the guy because, I, it's this also, that both have to enjoy it for it to be good, and yes…*

Swedish men were thought to be more liberal than men from cultures where FGC is performed, and thus likely to engage in sexual practices other than vaginal sex, such as oral sex. Fear of being exposed as cut and of being unable to enjoy oral sex made some women avoid dating Swedish men. Lola said: *Swedes are a bit more liberal and much more about oral sex and stuff like that, which is my absolute fear. If you're doing oral sex it's to stimulate the clitoris and I don't have that. I don't know if I consciously or unconsciously avoid them [Swedish men]…*

The women also believed that men with cultural backgrounds similar to their own would prefer non-cut women. Some recounted having been asked about their FGC status by new partners, with the underlying message that the man would end the relationship if FGC was confirmed. Yet, the men's disapproval of FGC was mostly related to infibulation; some of the women who had been defibulated and undergone clitoral reconstruction said they had told their new partner that they had “only been cut a little” (i.e., undergone less extensive forms of FGC), which seemed to be more accepted. However, while some women talked about being rejected based on their FGC, others talked about supporting, loving, and caring partners who expressed concern and empathy for their girlfriend or wife, including a wish for her to enjoy sex.

## Discussion

Almost all the interviewed women regarded the physical aspects of infibulation and clitorectomy as having harmed their sexual function, although they also acknowledged that psychological aspects of FGC affected their ability to enjoy sex. Sexual difficulties were perceived to cause struggles in their intimate relationships.

### Clitorectomy and its damage to sexual function

The women highlighted the physical aspects of clitorectomy as causing problems with sexual desire and sensation. This may not be surprising, as there is a growing body of literature supporting the importance of the clitoris for women's sexual function and orgasm (Levin, 2020; Limoncin et al., 2020; Mahar et al., 2020). Even in contexts where FGC is common, such as Somalia, the clitoris is commonly perceived as the physical site for women's sexual desire and pleasure, which is why it is seen as being in need of removal (Talle, 2007). At the same time, Somali women and men generally perceive types of FGC that remove all or parts of the external clitoris, commonly referred to as Sunna circumcision, as having few negative consequences for women's health and sexuality, at least compared to infibulation (Johansen, 2022). A disregard of the possible harm of clitorectomy on sexual function has also been demonstrated among researchers and healthcare workers (Dellenborg, 2004; Ahmadu, 2007; Ahmadu and Shweder, 2009; Jordal et al., 2020). Swedish gynecologists refuting the negative effect of the clitorectomy on women's sexual function (Jordal et al., 2020) highlight the internal structures of the clitoris, and thus perceive it impossible to “cut” the clitoris in any substantial way, as most of the clitoral organ will remain under the surface and be accessible to stimulation through the vagina (O'Connell et al., 1998). Healthcare providers and FGC scholars instead warn that an overemphasis of the physical consequences of FGC may become a self-fulfilling prophecy, causing women to anticipate their sexual function as “damaged” (Johnsdotter, 2018; Jordal and Griffin, 2018; Jordal et al., 2020; O'Neill et al., 2021). In contrast, cut women living in societies where FGC is highly regarded may perceive their sexual function positively, as suggested by Esho (2012) who studied FGC and sexual function among the Maasai people in Kenya. However, the women in our study opposed the construction of cut women's sexual problems as merely “psychological blocks”. Einstein (2008) discusses the possible biological effects of FGC on the brain and nervous system. She suggests that clitorectomy may involve a neurological rewiring in some women, which may explain why accounts of sexual function after FGC vary. Individual factors, as well as the extensive nature of the cutting (the clitoris glans, hood, bulb, etc.) and the fact that clitoral erectile tissue extends internally to a considerable degree, suggest that some cut women achieve orgasm through vaginal stimulation. On the other hand, as cutting the clitoris glans is likely to affect sensation both directly (by removing highly sensitive tissue) and indirectly (by cutting nerves connected to the inner portions of the clitoris and further altering sensation), other women may experience that their ability to feel sexual sensation and orgasm are reduced (Einstein, 2008). While it is difficult to distinguish between the physical and psychological factors involved with cut women's experiences of sex, future studies should aim to distinguish between various sexual practices as well as types and anatomical extents of FGC, and reconsider the possible biological consequences of clitorectomy.

### Sexual difficulties cause struggles in intimate relationships

The women in this study grieved their limited or excessive genital sensation, which they perceived as harming their ability to enjoy sexual activities and as causing struggles in their intimate relationships, which were all described as heterosexual. Some perceived an expectation to participate in penetrative sex to fulfill the man's needs and expectations in an intimate relationship, even if they themselves experienced a lack of desire or even discomfort and pain. Yet, an inability to enjoy sex was perceived to limit their partner's pleasure, which created shame and guilt. As the coital imperative is dominant within the heterosexual sexual script, with its implicit focus on child production (Levin, 2020; Limoncin and Nimbi, 2020; Mahar et al., 2020), penetrative sex is also often the focus in studies on the effects of FGC on sexual function (Obermeyer, 2005; Nour, 2006; Catania et al., 2007; Krause et al., 2011; Rouzi et al., 2017; Villani, 2022). However, due to criticism of the coital imperative, which has been shown to create an orgasm gap in heterosexual couples (Mahar et al., 2020; Andrejek et al., 2022), a new sexual script with increased focus on pleasure for both parties is likely to be on the rise. This is also illustrated in the narratives of the women in the present study on their perception of Swedish men being “more about oral sex”. Thus, expectations that they should enjoy sexual practices focusing on enhancing female pleasure, such as oral sex, seemed to pose additional stress for the interviewed women; not only because they felt they were “missing out” on desirable sexual experiences, but also due to a fear of failing to live up to gendered expectations of sexual enjoyment. Men from backgrounds similar to the women's own were also thought to value the woman's ability to enjoy sex, although they did not talk about them as being particularly concerned with oral sex. This could indicate a shift in perspective regarding women's sexuality even within cutting communities, which could be a driving force toward the eradication of FGC. However, the apparent contradiction between norms promoting Sunna circumcision to at least to some degree reduce women's sexual libido (Johansen, 2022) and men's desire for women to enjoy sex needs to be explored further. Nevertheless, a fear of failing to live up to expectations that they should enjoy sex made some women avoid intimate relationships, particularly with Swedish men. These findings suggest that cut women perceive themselves not to be “real women” in terms of contemporary ideals regarding female sexuality and gendered expectations and norms. Thus, new sexual scripts highlighting women's sexual pleasure may not be liberating for cut women, but may instead cause them to remain in the penetrative sexual script, as their FGC is less pronounced or noticed in such practices. Thus, we agree with Villani (2022) that future studies on FGC and sexual function need to include a broader spectrum of sexual practices than the heterosexual vaginal intercourse and the significance attributed to these practices.

### The importance of institutional recognition

While the interviewed women did not want to be recognized as “cut” by their partners and peers, they did want recognition by healthcare institutions and had all sought to undergo clitoral reconstruction. Gender scholar Ovesen (2020), who investigated help-seeking among lesbian victims of intimate partner violence (IPV), writes about the importance of institutional recognition. She suggests that there is an existing inequality in who receives institutional recognition (for example as a “victim of IPV”) and thus in who is considered worthy of protection and care and who is not. This renders some individuals' bodily needs unrecognized and unsupported, and thus more bioprecarious, than others' (Griffin and Leibetseder, 2020; Ovesen, 2020). Recognition, Ovesen argues, is not only about who is counted as a victim; it also concerns individuals' sense of belonging within a certain context. In the present study, institutional recognition could be translated into the offering of clitoral reconstruction. Clitoral reconstruction, while growing in popularity, is still not available in most countries (Jordal and Griffin, 2018; Villani, 2022). While there are currently no recommendations supporting clitoral reconstructive surgery from mainstream medical bodies such as the WHO and the RCOG in the UK (Royal College of Obstetricians and Gynaecologists, 2015; WHO, 2016; Villani, 2022), which could be related to a fear of exposing cut women to unnecessary surgical risks and pain (Bah et al., 2021), many women who have undergone clitoral reconstruction claim it has helped them gain a newfound ability to enjoy sex (including oral sex) or to now no longer feel “cut” and thus less ashamed and distressed in intimate relationships (author).

Sexuality is embedded in power relations, many of which are gendered (Villani, 2022). The interviewed women's request for clitoral reconstruction could be seen as a desire to transgress the boundaries of the coital imperative, which is increasingly portrayed as insufficient for achieving the full possibility to experience sexual pleasure. It can also be seen as a desire to balance out existing power differences whereby cut women are regarded as inferior, in being judged not only as “cut” in a context in which FGC is considered “barbaric and backwards” (Pred, 2000; Pedwell, 2010) but also as incapable of the full possible experience of sexual enjoyment (Jordal et al., 2018; Villani, 2018). In a Norwegian study, the authors demonstrated that women with more liberal attitudes regarding gender and sexual equality were also more positive to seeking out FGC-related healthcare (Ziyada et al., 2020). This could indicate that cut women seeking help for sexually related problems in Sweden are also those who have taken up the host country's ideals of gender equality and sexual rights, an indication of societal and ideological integration. At the same time, choosing reconstructive clitoral surgery to integrate in the host society may involve new concerns for the women involved.

### Methodological considerations

All the interviewed women in this study had sought to undergo clitoral reconstruction. Many women requesting this surgery in Sweden hope to, at least partly, improve their sexual function (author). Thus, the choice of recruitment may cause selection bias as this group of women may attribute greater importance to the clitorectomy for their self-experienced sexual problems than women who do not seek clitoral reconstruction. Thus, more research investigating perceptions and experiences of FGC and sexual function among cut women who do not seek out clitoral reconstruction is needed. At the same time, one cannot assume that women who do not request surgery have different perceptions and experiences. As suggested by Ziyada et al. (2020), variations in healthcare seeking are not necessarily due to differences in experiences; instead, they may reflect differences in the perceived need to improve their sexual function or willingness to break with social norms. That many of the interviewed women worked in healthcare (as nurses, nurse assistants, or midwives) might also indicate that the interviewed women were aware of available healthcare interventions to a higher degree than those not working in this field.

Disentangling the physical and psychological aspects of the connection between FGC and sexual function is difficult, if not impossible. Therefore, this was not the objective of the present study; rather, we wanted to explore the multifaceted ways in which women reason around the potential connection between FGC and sexual function. While the women were asked about why they had requested clitoral reconstruction, the connection they perceived between FGC and sexual function was not a major theme during the interviews. Rather, the interviews' primary purpose was to understand the women's motives and expectations for the surgery and their experiences of its after-effects. One could therefore assume that more profound answers would have emerged if the interviews had been dedicated to exploring interlinkages between FGC and sexual experiences. Nevertheless, our choice to let the women recount sexual aspects when examining their motives for surgery, as well as its after-effects, resulted in a wide diversity of reflections on the matter. We chose this approach to avoid causing the study participants any discomfort, even though it may have prevented us from uncovering detailed information, particularly on self-experienced sexual problems. Yet, the fact that the interviewer did not push the women to talk in detail about their sexual experiences also means that the accounts of sexual difficulties were largely self-derived. We believe that this was a sound compromise, not only because the data still contains valuable accounts on both general matters and personal experiences, but more so because we find that the woman's well-being and integrity in the interview situation are more important than pushing her to speak about difficult matters. Also, that the women were not pushed likely means that the issues that came up were something they had reflected on beforehand and were not merely a reality created in interaction with the interviewer.

## Conclusion

The women interviewed for this study understood clitorectomy as having damaged their sexual function, which they felt had negatively affected their intimate relationships. While not rejecting the notion that psychological aspects of FGC were also reducing their ability to enjoy sex, they wanted the physical consequences of FGC on their sexual function to be recognized as “real” and not be dismissed or explained away as “psychological blocks”. Future studies on FGC and sexual function need to consider the complexity of the psychological, physiological, and socio-cultural-symbolic aspects of FGC and include a broader spectrum of sexual practices than heterosexual intercourse and the significance attributed to them.

## Data availability statement

The datasets presented in this article are not readily available because the data is considered of sensitive nature. Requests to access the datasets should be directed to MJ, malin.jordal@hig.se.

## Ethics statement

The studies involving human participants were reviewed and approved by Regional Ethical Review Board in Stockholm. Written informed consent for participation was not required for this study in accordance with the national legislation and the institutional requirements. The patients/participants provided their oral informed consent to participate in this study.

## Author contributions

All authors listed have made a substantial, direct, and intellectual contribution to the work and approved it for publication.

## Funding

The University of Gävle covered the costs for open access and English proof reading of the manuscript.

## Conflict of interest

The authors declare that the research was conducted in the absence of any commercial or financial relationships that could be construed as a potential conflict of interest.

## Publisher's note

All claims expressed in this article are solely those of the authors and do not necessarily represent those of their affiliated organizations, or those of the publisher, the editors and the reviewers. Any product that may be evaluated in this article, or claim that may be made by its manufacturer, is not guaranteed or endorsed by the publisher.
